# Causes and outcome of pregnancy related acute kidney injury

**DOI:** 10.12669/pjms.40.1.7444

**Published:** 2024

**Authors:** Noor Muhammad, Nazia Liaqat

**Affiliations:** 1 Noor Muhammad, MBBS, MCPS, FCPS Assistant Professor, Department of Nephrology. Medical Teaching Institute, Lady Reading Hospital, Peshawar, Pakistan; 2 Nazia Liaqat, MBBS, FCPS Assistant Professor, Department of Gynecology. Medical Teaching Institute, Lady Reading Hospital, Peshawar, Pakistan

**Keywords:** Pregnancy related acute kidney injury, Obstetrical causes, Renal outcome

## Abstract

**Objective:**

To determine frequencies of causes and renal outcomes of pregnancy related acute kidney injury.

**Method:**

This descriptive case series study was conducted in Nephrology unit of a tertiary care hospital of Peshawar, from 1^st^ August 2021 to 31^st^ July 2022.A total of 100 patients with acute kidney injury secondary to obstetric conditions were enrolled via non-probability consecutive sampling technique. While patients with pre-existing renal disease, those with renal stones, or having bilateral small kidneys on ultrasound were excluded from the study. Patients were followed till 12 weeks postpartum period. Underlying obstetrical causes and outcome at 12 weeks postnatal period were determined.

**Results:**

The mean age of sample of 100 cases was 29.29 ± 6.45. Mean serum creatinine at presentation was 6.5± 3.13. Majority of patient, 89% were multigravidas. Seventy eight percent patients required hemodialysis. Primary postpartum hemorrhage remained the commonest underlying cause of pregnancy related acute kidney injury in this study. The frequency of persistent renal failure in Pr-AKI (pregnancy related acute kidney injury) in this study was 14%. In about 66% of cases complete recovery occurred. All the underlying obstetrical causes, when adjusted for age, gravidity, place and mode of delivery, had no association with persistent renal failure.

**Conclusion:**

Primary postpartum hemorrhage is the predominant cause of pregnancy related acute kidney injury. By the end of 12 weeks postpartum, two third patients recover completely from pregnancy related acute renal injury.

## INTRODUCTION

Pregnancy related acute kidney injury (Pr-AKI) is a potential cause of fetomaternal morbidity and mortality worldwide.1 Pr-AKI results from conditions specific to pregnancy. In pregnancy, the common causes of acute kidney injury are preeclampsia, hemorrhage and sepsis.2 The incidence of Pr-AKI is estimated to be 2% on average, around the globe.^3^ A substantial decline in the incidence of Pr-AKI has occurred, in the developed countries. This is attributed to strong and comprehensive antenatal care programmes.

On the contrary, situation inthe underdeveloped world is devastating, where preeclampsia, hemorrhage and sepsis continue to remain the killer conditions in pregnancy. The survivors of these conditions suffer serious morbidities, of which kidney injury is the most common one. The incidence in countries like Pakistan varies from 0.02 to 71%.3 In low resource countries the major contributors to Pr-AKI are hemorrhage and sepsis. In high resource countries hypertensive disorders of pregnancy, thrombotic thrombocytopenic purpura and hemolytic uremic syndrome have largely replaced hemorrhage and sepsis as causes of Pr-AKI.3

The independent risk factors for Pr-AKI, quoted in literature are older age ,history of preeclampsia and diabetes.4 The predisposing factors for hemorrhage, sepsis and preeclampsia can generally be considered as risk factors for Pr-AkI. These include lack of dependable antenatal care, extreme of maternal ages, anemia in pregnancy, multifetal gestation, prior unfavorable pregnancy outcome, and intrauterine fetal demise.5 There is no standardized clinical or laboratory diagnostic criterion for Pr-AKI. The commonly used Risk, Injury, Failure, Loss, End stage renal disease (RIFLE) and Acute Kidney Injury Network (AKIN) criteria are not validated so far for use in pregnancy.6 Hence different criterion have been used in different studies.

Pr-AKI is associated with serious complications for both mother and fetus. Fetomaternal complication mainly result from the underlying cause of acute kidney injury. Preterm delivery, intrauterine fetal death, still births, intensive care admissions of both mother and baby, prolonged hospital stay and maternal mortality are the documented obstetrical complications.7

While in majority of patients who survive the deadly obstetrical complications, renal recovery occurs. Recovery from renal injury is dependent on timely diagnosis and appropriate management of underlying condition as well as of renal injury. This current study was planned with the intent to determine the frequencies of causes and renal outcomes of pregnancy related acute kidney injury. This study will provide an insight into the burden of this reversible health condition resulting from potentially preventable obstetrical complications. It will also open window for further larger studies in this field.

## METHODS

This descriptive case series study was conducted in the departments of Nephrology from 1^st^ August 2021 to 31^st^ July 2022. Sample size was calculated using OpenEpi online sample size calculator. For calculation of sample size confidence level was set at 95% and proportion of persistent renal failure was taken as 6.7%.^8^

### Ethical approval

It was taken from the ethical review board of the hospital (Ref#188/LRH/MTI dated:120-07-2021). A total of 100 patients meeting the eligibility criterion were enrolled into the study, after taking informed consents. Patients were enrolled via nonprobability consecutive sampling technique.

Inclusion & Exclusion Criteria.Women with acute kidney injury secondary to obstetric complications at term, were included in this study. While patients with preexisting renal disease, those with renal stones, or having bilateral small kidneys on ultrasound were excluded from the study. Renal failure was defined according to AKIN criterion as having any of the following; (1) rise in serum creatinine by 0.3 mg/dl or more in fourty eight hours, (2) increase in serum creatinine to 1.5 times or more of baseline levels within the last seven days, (3) urine output of less than 0.5 ml/kg/hour for six hours.9 Persistent renal failure was defined as cases who were still dialysis dependent at 12 weeks postnatal follow up time. Complete recovery was defined as return of serum creatinine levels to normal by 12 weeks postpartum period. Causes for Pr-AKI were defined as the sole primary obstetrical event leading to AKI.

Detailed history was taken from each included patient regarding their antenatal course, including history of reliable and regular antenatal care, presence of pregnancy specific conditions like pregnancy induced hypertension, gestational diabetes, anemia, antepartum hemorrhage. History of intrapartum events like mode of delivery, fetal outcome, post- partum hemorrhage, blood transfusions was also taken. All these patients were followed till 12 weeks postpartum period. Patients who were discharged home on conservative management plan were advised and encouraged to visit outpatient department or Nephrology ward for follow ups. Those who failed to turn up at their own were contacted via phone calls and encouraged to come.

Data was analyzed via SPSS version 25. Frequencies and percentages were calculated for categorical variables and mean, and standard deviations were computed for numerical variables. Chi square test was applied to find out associations between categorical variables. Independent sample t-test was used to compute mean differences of numerical variables between two groups. For variable showing significant associations multivariate logistic regression analysis was carried out to determine strength of associations after controlling for confounders. (Adjusted odd ratios)

## RESULTS

**Fig.1:**
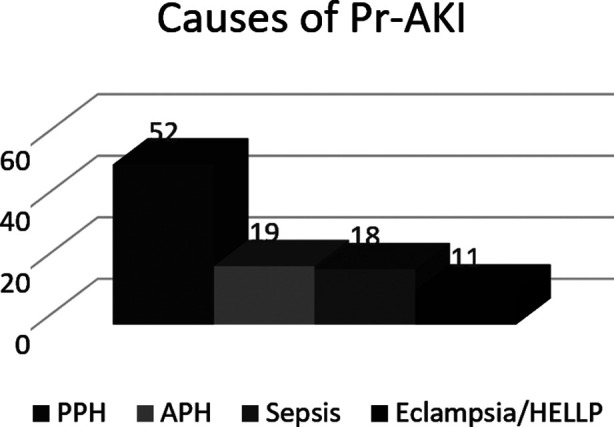
Frequencies of causes of Pr-AKI.

The mean age of sample of 100 cases was 29.29 ± 6.45. Mean serum creatinine at presentation was 6.5± 3.13. Thirty four percent patients were less than 26 years of age. Majority of patient, 89 % were multigravidas. Regular and reliable antenatal care history was present in only 30% of patients. History of massive transfusion was present in 54% cases. Intrauterine fetal death was present 49% cases whereas neonatal death occurred in 6% cases. Seventy eight percent patients required hemodialysis. Primary postpartum hemorrhage remained the commonest underlying cause of pregnancy related acute kidney injury in this study, (Fig.1).

The frequency of persistent renal failure in Pr-AKI in this study was 14%. In about 66% of cases complete recovery occurred. Seven percent of patients had partial recovery. Loss to follow up occurred in 13 cases. The underlying obstetrical causes did not have significant associations (p>0.05) with persistent renal failure. (Table-I).

**Table-I:** Association of underlying causes with persistent renal failure.

Causes	Persistent Renal Failure		Missing	Row total	p-value

	YES	NO			
PPH	07	38	07	52	0.88
APH	03	12	04	19	0.55
Sepsis	01	16	01	18	0.87
Eclampsia/HELLP	03	07	01	11	0.77
Column total	14	73	13	100	

## DISCUSSION

According to this study primary postpartum hemorrhage is the predominant cause of pregnancy induced acute kidney injury. This study showed frequency of 14% for persistent renal failure in cases of pregnancy related acute kidney injury. It further proved that persistent renal failure has no association to underlying obstetrical causes when controlled for other factors. The mean age of study population in this study was 29.29 ± 6.45. This is consistent with the findings determined by other studies from this region of the world.^2^,^7^ This is because of early marriages in this part of the world and delaying pregnancy to later ages in industrialized countries.

It is also reinforcing the fact of increased burden of deadly obstetric complication leading to acute kidney injury at relatively younger age compared to high resource countries. This is attributed to lack of structured and effective antenatal care programmes in low resource countries. The mean age of study population in other studies from Pakistan done by Hasan et al.10 and Naqvi et al.^11^ was 25±6.1 and 28.53±6.7 respectively. The study by Naqvi et al has discussed the role of various biomarkers in prediction of AKI in high risk obstetric population. Other various Indian studies also reported acute kidney injury in pregnant population at younger age.^12^,^13^ Regarding parity,89% of patients in this study were multigravidas ,this is in contrast to study done by Mahesh in India.^12^

Primary postpartum hemorrhage was the predominant underlying cause of acute kidney injury accounting for 52% cases, in the current study. In a similar study done in Pakistan ,back in 2009 ,postpartum hemorrhage was the pre dominant cause of pregnancy related AKI.10 Thirteen years after, this potentially preventable condition is still the principal cause of Pr-AKI in Pakistan, questioning the effectiveness of health care programmes. Other studies done in the region have reported similar burden of postpartum hemorrhage in cases of Pr-AKI.^13^ In a study done in Nigeria PPH accounted for 50% cases of Pr-AKI.^14^ Similarly in an Egyptian study primary postpartum hemorrhage was one of the commonest cause of Pr-AKI.^15^

In this study antepartum hemorrhage and sepsis were the second and third common causes of Pr-AKI with nearly equal percentages of 19% and 18% respectively. This finding about antepartum hemorrhage is similar to those reported in the study done by Hasan in Pakistan.^10^ However relatively higher percentages of antepartum hemorrhage have been reported in certain other studies. Like study done by Sahay reported 30% contribution of antepartum hemorrhage to cases of Pr-AKI.^13^ Whereas in the study done by Prakash, antepartum hemorrhage was found in 8.3% cases of Pr-AKI.^8^

In the current study percentage of sepsis was fairly low compared to study done by Prakash et al. in India, who reported 25.8% cases of sepsis in Pr-AKI.^8^ In another Indian study sepsis accounted for almost half of the cases of Pr-AKI.^16^ The author of this Indian study has also raised concerns about the unusual high rates of sepsis in their study despite an increased number of institutional deliveries. These differences can be due to the reason that in the current study, patients who developed AKI due to late pregnancy complications were enrolled.

In the current study eclampsia/HELLP was least common cause of Pr-AKI, accounting for 11% cases. This finding is similar to another local study.^10^ However the reported proportion of eclampsia in the current study is very low compared to many other studies where the stated percentages are 35%,^17^ 44.5%,^13^ 56%,^12^ and 73%.^18^ These high rates are basically due to reduction in number of cases of postpartum hemorrhage attributed to advanced health systems in contrast to high prevalence of obstetrical hemorrhage in the region of current study.

The frequency of persistent renal failure was 14%, this is significantly high compared to 2.4% cases of dialysis dependent end stage renal failure cases, reported in a systematic review and meta-analysis.19 In the current study patients were followed only till 12 weeks postpartum, longer follow ups can detect the number of end stage renal failures. In this study, complete recovery was observed in 66% cases. This recovery percentage is better than 56% reported in an Indian study.20 In the same study persistent renal failure occurred in 36% cases, which is quite high compared to 14% determined by the current study.

### Limitations

This was a prospective case series. However, it was a single center study with no control group and short term follow ups.

## CONCLUSION

Primary postpartum hemorrhage is the predominant cause of pregnancy related acute kidney injury. By the end of 12 weeks postpartum, two third patients recover completely from pregnancy related acute renal injury.

### Author’s Contributions:

**NM:** Concepts, Literature search, Data acquisition, manuscript editing

**NL:** Clinical studies, Data analysis, Design, Literature search, Manuscript Preparation.

**NM,** **NL:** Responsible and accountable for the accuracy and integrity of the work.
